# A Grand Lecture

**Published:** 1877-05

**Authors:** 


### *A GRAND LECTURE.*

It was a grand lecture on a grand subject, to which we listened on Washington's Birthday at Chickering Hall. The speaker was Ex-Governor Fuller, and the subject was "The True Philosophy of Digestion." There were salient hits at the follies of our alimentary system, together with a recital of the better modes indicated by modern science. As a matter of course, the fallacy of the bran-bread theory of Graham was exposed, because science discovers no physiological argument in favor of the use of wholly insoluble food substances such as Graham advocated. The audience embraced large numbers of our best citizens, including doctors, divines, lawyers, judges, and bankers, and we never heard more cordial and enthusiastic applause than was bestowed upon the eloquent speaker. For the sake of humanity we wish this admirable lecture could be heard in every hall and church in the land.

---

Ormon's Magic Cleanser.—In our Journal for March, 1876, we spoke favorably of an article which we had tested for the removal of spots and stains from clothing. We were led to try it by our tailor, who confided to us the fact that all the best city tailors were in the habit of employing the fluid alluded to to cleanse and freshen soiled clothes. Everybody knows how hard it is to keep clothes, especially coat and vest fronts, from becoming soiled, and how nearly useless soaps and benzines are in the removal of such defacements. With this article there is not much trouble in keeping one's clothing neat and fresh and free from spots. It is now sold by the Health Food Company and by Macy in New York, and by Loesser in Brooklyn. The office of the proprietor, Mr. H. Wiswell, is with Lipe, Nearing & Co., 54 Lispenard Street, New York.

